# Cerebellar volume measures may differentiate multiple sclerosis fallers from non-fallers

**DOI:** 10.21203/rs.3.rs-4213155/v1

**Published:** 2024-04-19

**Authors:** Taylor N. Takla, Jennie Feldpausch, Erin M. Edwards, Shuo Han, Peter A. Calabresi, Jerry Prince, Kathleen M. Zackowski, Nora E. Fritz

**Affiliations:** Wayne State University; Wayne State University; Wayne State University; Johns Hopkins University; Johns Hopkins University; Johns Hopkins University; Johns Hopkins University; Wayne State University

**Keywords:** Multiple sclerosis, falls, cerebellum, cognition, motor functioning

## Abstract

**Introduction:**

The cerebellum is a common lesion site in persons with multiple sclerosis (PwMS). Physiologic and anatomic studies have identified a topographic organization of the cerebellum including functionally distinct motor and cognitive areas. This study implemented a recent parcellation algorithm developed by Han et al., 2020 to a sample of PwMS and healthy controls to examine relationships among specific cerebellar regions, fall status, and common clinical measures of motor and cognitive functions.

**Methods:**

Thirty-one PwMS and 29 age and sex-matched controls underwent an MRI scan and motor and cognitive testing. The parcellation algorithm was applied to all images and divided the cerebellum into 28 regions. Mann-Whitney U tests were used to compare cerebellar volumes among PwMS and controls, and MS fallers and MS non-fallers. Relationships between cerebellar volumes and motor and cognitive function was evaluated using Spearman correlations.

**Results:**

PwMS performed significantly worse on functional measures compared to controls. We found significant differences in volumetric measures between PwMS and controls in the corpus medullare, lobules I-III, and lobule V. Volumetric differences seen between PwMS and controls were primarily driven by the MS fallers. Finally, functional performance on motor and cognitive tasks was associated with cerebellar volumes.

**Conclusions:**

Using the parcellation tool, our results showed that volumes of motor and cognitive lobules impact both motor and cognitive performance, and that functional performance and cerebellar volumes distinguishes MS fallers from non-fallers. Future studies should explore the potential of cerebellar imaging to predict falls in PwMS.

## Introduction

Multiple Sclerosis (MS) is a progressive neurodegenerative disease [1]. MS-driven pathology (i.e. demyelination and axonal damage) disrupts neural communication across the central nervous system and leads to a wide clinical spectrum of debilitating motor [2], cognitive [3], and sensory impairments [4]. The cerebellum is a commonly impacted brain region in MS, and individuals with MS often experience cerebellar dysfunction, contributing to motor, cognitive, and sensory deficits. As a result, persons with MS (PwMS) are at an increased risk for injurious falls [5], [6] that reduce quality of life [7]. Common fall risk assessments in PwMS include forward walking speed and balance measures, however these measures are limited in their predictive validity to detect falls [8].

Our lab has demonstrated associations between neuroimaging metrics and fall risk [9], including the utility of combining clinical measures with quantitative imaging metrics to improve identification of fallers in PwMS [10–11]. Given the complexity and diversity of factors associated with fall risk, it is likely that several brain regions are contributing to fall risk as well. Although our prior work has identified corticospinal tract and superior cerebellar peduncles as important white matter tracts contributing to fall risk, additional regions of the cerebellum have also been linked to fall risk in PwMS [12–13]. Furthermore, the cerebellum is a common lesion site in PwMS, and cerebellar pathology has been associated with debilitating functional deficits [14] across multiple domains disrupted by MS, including motor, sensory, and cognitive processes [15], all of which contribute to fall risk. Additionally, MRI studies have shown cerebellar volumetric abnormalities contribute to disability and performance on cognitive tasks [16].

Physiologic and anatomic studies in healthy adults and other clinical populations have identified a topographic organization of the cerebellum which includes discrete motor and cognitive areas [17], with motor areas linked to posture [18] and gait coordination [19], and cognitive areas linked to information processing, working memory and visuospatial memory [17]. Our laboratory has recently showed that these functionally distinct motor and cognitive regions of the cerebellum correlate strongly with both motor and cognitive function among persons with MS, supporting the role of the cerebellum in both motor and cognitive processes [10]. However, the development of a recent cerebellar parcellation algorithm using deep network methods, which is shown to be superior in a cerebellar parcellation challenge [20], has not yet been applied to the MS population. Applying this parcellation algorithm, which has demonstrated state of the art performance [21], offers a novel opportunity to better understand specific cerebellar structure-function relations by examining the relation of cerebellar volumetrics to fall risk and motor and cognitive functioning in PwMS.

This present study aimed to implement this recent cerebellar parcellation algorithm [21] to our sample of PwMS and healthy controls in a secondary analysis of volumetric measures of the cerebellum [10]. We hypothesized that 1) MS and Control (HC) groups would show no significant differences in cerebellar volume measures; 2) individuals who report a fall within the past month (fallers) would perform worse on measures of motor and cognitive function and would have lower cerebellar volumes than individuals who report no falls within the past month (non-fallers); and 3) volumetric measures of the motor and cognitive cerebellar lobules would be associated with motor and cognitive task performances, respectively. Application of a recent, innovative parcellation scheme to the cerebellum, a complex brain region that is commonly affected in people with MS and is associated with a diverse range of impairments, is critical to better understand how cerebellar involvement impacts functioning and falls. Through this approach, we aim to ultimately provide insights that can guide targeted rehabilitation therapies for cerebellar dysfunction in PwMS.

## Materials and Methods

Individuals with relapsing remitting multiple sclerosis (RRMS) and healthy age and sex-matched controls enrolled in a larger intervention trial were recruited for this study. PwMS were included if they were ambulatory with or without an assistive device. Participants were excluded if they had experienced a MS relapse within three months of testing, reported corticosteroid use within 30 days of testing, or reported a history of orthopedic or neurologic conditions that might interfere with testing procedures. All participants were able to follow study-related commands and gave written informed consent. The Institutional Review Boards at both Johns Hopkins Medical Institute and Kennedy Krieger Institute approved the study procedures. All research was carried out in accordance with the 1964 Declaration of Helsinki and its later amendments.

In a single session, demographic information [age, sex, fall history, symptom duration, and Expanded Disability Status Scale (EDSS)], patient-reported outcomes of pain, fatigue and quality of life, cognitive function, and quantitative measures of strength, sensation, lower extremity coordination, balance, and walking were collected. Fallers were classified as individuals who reported at least one fall within the prior month. Neuroimaging measures were collected within three weeks of this session. Control participants completed all measures with exception of MS-specific assessments.

### Motor Measures

Timed Up and Go (TUG). Participants were instructed to stand from a chair, walk 10-feet, turn, walk back, and return to a sitting position in the chair at their quickest and safest speed without running [22]. The TUG is reliable and incorporates dynamic balance during functional tasks of turning, transitioning, and walking [23].

#### Timed 25 Foot Walk (T25FW).

Participants were instructed to walk at their quickest, safe speed along a flat 25-foot walkway [24]. Participants completed two walking trials, with the final score calculated as the average of the two trials. The T25FW has established reliability [25] and validity [26].

#### Walk Velocity.

Participants were instructed to walk at their quickest, safest speed across a 20-foot Zeno Walkway (Protokinetics, Havertown, PA, USA), which records footfalls in real-time. Participants completed six walking trials across the mat. Average walk velocity for each individual was calculated using a custom MATLAB program (The MathWorks, Inc., Natick, MA, USA).

#### Two-Minute Walk Test (2MWT).

Participants were instructed to cover as much distance as possible while walking for two minutes. The 2MWT has established reproducibility and reliability in MS [27].

#### Strength Assessment.

Maximal voluntary contraction of bilateral hip flexion, hip extension, and hip abduction was assessed with a handheld dynamometer (Hoggan Health Industries, West Jordan, UT, USA) using previous methods from our laboratory [28]. The average of two trials of each muscle was recorded and bilateral strength summed. Quantitative strength testing is reliable and valid for PwMS [29].

#### Six Spot Step Test (SSST).

Participants were instructed to walk as quickly and safely as possible in a crisscross pattern to six spots along a five-meter pathway. Each “spot” includes a weighted box that participants kicked away from its original position using the medial and lateral sides of their foot. Participants used only one foot to kick all six weights, and time was recorded. Participants performed two trials with each leg, and the average time of four trials was the final score [30]. The SSST requires lower extremity coordination and is validated in PwMS [31].

#### Romberg Balance Assessment.

Participants were asked to balance in six different positions adapted from the Romberg and Sharpened Romberg tests [32–33]. To progress to the next condition, participants had to stand independently for 30-seconds in the prior condition. Scores were tallied for the number of successful conditions (maximum score of six), including feet apart-eyes open, feet together-eyes open, feet apart-eyes closed, feet together-eyes closed, feet in tandem-eyes open, feet in tandem-eyes closed. Participants placed their feet shoulder-width apart during feet apart conditions.

### Sensory Measures

#### Sensation Assessment.

Sensation was quantified bilaterally at the great toe using a Vibratron II device (Physitemp, Huron, NJ, USA). The Vibratron provides reliable and objective quantitative measures of vibratory sensation in PwMS [29] and may be used as a proxy measure for proprioception [34]. Participants identified which of two rods was vibrating, and the threshold [35] from the worse toe was calculated and used for data analysis.

### Fall Assessment

#### Fall Assessment.

Participants self-reported a one-month fall history. A fall was defined as an unintentional event that resulted in the person hitting the ground.

### Cognitive Measures

#### Symbol Digit Modalities Test (SDMT).

Participants received a key with nine numbers each corresponding to a symbol and were asked to determine the number belonging with a series of symbols using this key. The score is the number of correct answers in 90 seconds. The SDMT is a validated and reliable test in MS to analyze information processing speed [36] and is recognized as the single best measure to assess cognition in PwMS under time constraints [37].

### Patient-Reported Outcomes

*Multiple Sclerosis Walking Scale-12 (MSWS-12)* was used to examine self-reported walking dysfunction [38]; the *Brief Pain Inventory (BPI)* [39–40] was used to examine pain severity and interference; and the *Multiple Sclerosis Quality of Life (MSQoL)* [41] and *Short Form-36 (SF-36)* [42–43] were used to examine health-related quality of life. The MSQoL has mental, physical and fatigue subscales, while the SF-36 has only mental and physical subscales.

### Structural Magnetic Resonance Imaging (MRI) Acquisition

All participants participated in whole-brain imaging collected on the same 3-Tesla Intera scanner (Philips Medical Systems, Best, The Netherlands). Two axial whole-brain sequences were acquired, a T2-weighted fluid-attenuated inversion recovery (FLAIR; acquired resolution: 0.9×0.9×1.0mm; TE: 365ms; TR: 4.8s; TI: 1.6s; SENSE factor:1); and a 3D magnetization-prepared rapid acquisition of gradient echoes (MPRAGE; acquired resolution: 0.8×0.8×1.2mm; TE: 6ms; TR: 10ms; flip angle:8°; SENSE factor:1). Last, 32-direction diffusion-weighted images were acquired at an isotropic resolution of 2.2mm.

### Image Analysis

Following the methods of Han et al. 2020, we applied the ACAPULCO cerebellum parcellation algorithm based on convolutional neural networks [21, 44] to all images. Briefly, the images were inhomogeneity-corrected using N4 [45] and rigidly registered to the 1 mm isotropic ICBM 2009c template [46] in MNI space using the ANTs registration suite (http://stnava.github.io/ANTs/). To perform per-voxel labeling, parcellation classifiers from 15 expert manual delineations [20] were used. The result of this algorithm was 28 cerebellar regions: bilateral lobules I–III, IV, V, and VI; crus I and II; lobules VIIB, VIIIA, VIIIB, IX, and X; vermis VI-X; and corpus medullare. To examine the relationship of motor and cognitive function with cerebellar volumes, we examined lobules I-V and VIII, which have been linked with motor function, and lobules VI-VIIB and crus I-II (part of lobule VII), which have been linked with cognitive function [17].

### Statistical Analyses

Data analysis was performed using IBM SPSS Statistics V 28.0.1.0. Mann-Whitney U tests were used to compare cerebellar volumes among PwMS and matched controls, and between MS fallers and MS non-fallers. We observed no significant differences in volume (all *p* > 0.05) between right and left cerebellar structures, thus combined bilateral values were used for all analyses. A Spearman correlation was used to assess the relationship cerebellar volumes to performance on clinical functional outcome measures (e.g., motor and cognitive measures). To understand the overall relationship of functional measures to average bilateral lobule volumes, we combined the MS and matched controls into a single group and utilized Spearman correlations.

## Results

### Participants

Thirty-one PwMS and 29 healthy age and sex-matched controls participated in this study. There were no significant differences between PwMS and controls for age or sex. Of the PwMS, 15 were identified as fallers (≥1 fall in the past month) and 16 were identified as non-fallers (0 falls in the past month). Notably, our MS group had low disability and was highly ambulatory (mean EDSS of 3.8). Further demographic information is presented in Table 1.

### Functional Performance in MS and HC

PwMS performed significantly worse on motor measures compared to HCs. Specifically, PwMS had a slower time to complete the TUG and T25FW, slower walking velocity, a shorter distance walked on the 2MWT, decreased summed strength, slower performance on the SSST, poorer balance, and worse vibration sensation (all *p* < 0.05). PwMS also performed significantly worse on the SDMT, had greater pain severity and interference, as well as worse mental and physical quality of life on the SF-36 compared to controls (all *p* < 0.05), see Table 1.

### Cerebellar Volume Measures in MS and HC

HCs exhibited significantly greater volume measures in the corpus medullare, lobules I-III, and lobule V when compared to PwMS (Supplementary Table 1). There was no significant difference in the other individual lobules, Crus I and II, or the combined motor and cognitive lobule volumes between PwMS and HCs (see Supplementary Table 1).

### Relationships between Cerebellar Volume and Clinical Function

#### Motor and Sensory Measures

**Figure 1:**
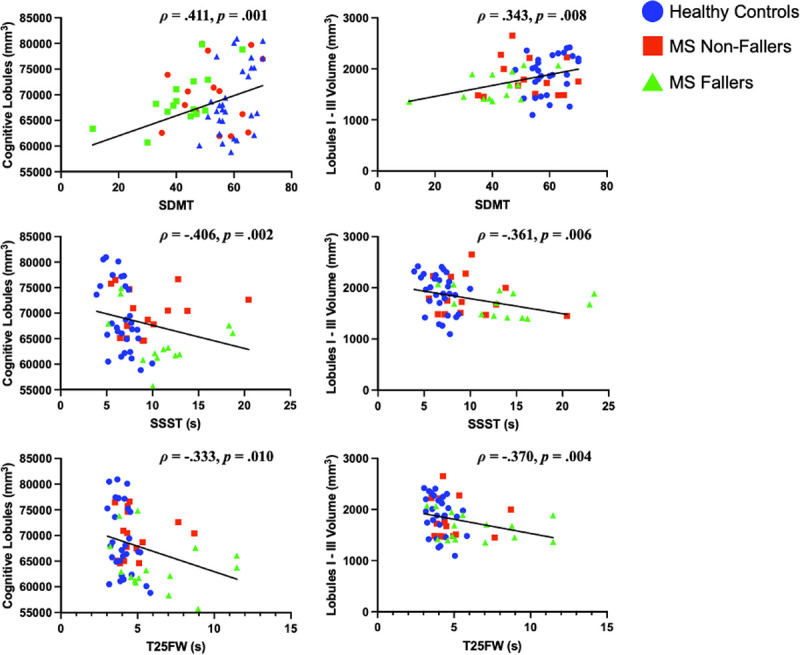
Scatterplots showing the relationships between cerebellar volumes and clinical function Fig 1 All correlation values listed are Spearman’s rho (ρ). Symbol Digit Modalities Test (SDMT); Six Spot Step Test (SSST); Timed 25 Foot Walk (T25FW); lobules I-III are generally considered motor lobules. Cognitive lobules indicate the sum of lobules VI-VII and crus I-II. Volumes of both motor and cognitive lobules were correlated with performance on clinical measures of motor and cognitive functioning. Figure was created with GraphPad Prism 10

Lower volume of lobules I-III was associated with significantly longer times to complete the TUG, the T25FW and the SSST, slower walking velocity, worse strength, poorer balance, and higher (worse) vibratory threshold (see Figure 1 and Table 2). Lower volume of lobule V was associated with poorer balance (Table 2), but no other motor measures. There were no significant relationships among function and other motor lobules (lobules IV, VIIIA, VIIIB).

Lower volumes in lobules VI and crus I were significantly associated with slower time to complete the T25FW and SSST, slower walking velocity, and worse strength (Table 2). Lower Crus I volume was also significantly correlated with slower time to complete the TUG, shorter distance covered by the 2MWT, and poorer balance (Table 2). Lower volume in crus II was associated with significantly reduced walking velocity and longer time to complete the SSST, but no other measures. There were no significant relationships among function and the other cognitive lobule (lobule VIIB).

Interestingly, lobule IX also demonstrated significant associations with motor functions, with lower volume significantly correlated with slower time to complete the TUG, the T25FW, the SSST, slower walking velocity, less distance covered in the 2MWT, and worse strength (Table 2).

#### Cognitive Measures

Poorer performance on the SDMT was associated not only with lower volumes in cognitive lobules VI and crus I, but also with reduced volume in lobules I-III, which are typically characterized as motor lobules (Figure 1, Table 2).

#### MS fallers vs. non-fallers

As expected, MS fallers performed significantly worse than MS non-fallers on the SDMT and reported lower scores on measures of quality of life (excluding SF-36); however, there were no significant differences between fallers and non-fallers on any motor measures (Table 1). There was no significant difference between non-fallers and HC on waking velocity, 2MWT, pain severity, or SF-36 mental sub score, while fallers performed significantly worse than HCs on these measures (Table 1). Significant differences in MS fallers and MS non-fallers volume measures were found at the corpus medullare (*p* = 0.027), lobule VI (*p* = 0.041), vermis IX (*p* = 0.033), vermis X (*p* = 0.017) and in the combined cognitive lobules (*p* = 0.007) (Table 3). When compared to HCs, MS fallers demonstrate significantly reduced volume in the corpus medullare (*p* = 0.013), lobules I-III (*p* = 0.024), lobule V (*p* = 0.012), lobule VI (*p* = 0.009), crus I (*p* = 0.046), vermis X (*p* = 0.032) and cognitive lobules (*p* = 0.022). Interestingly, there were no significant differences in cerebellar volumes between MS non-fallers and controls, suggesting that the difference between MS and control groups may be driven by the fallers (Table 3). Further, when all non-fallers are combined into a single group (including both HC non-fallers and MS non-fallers) and compared to the group of MS fallers, fallers demonstrates significantly reduced volume of corpus medullare (*p* = 0.008), lobules I-III (*p* = 0.030), lobule V (*p* = 0.035), lobule VI (*p* = 0.007), crus I (*p* = 0.047), vermis X (*p* = 0.012), and the cognitive lobules (*p* = 0.006) (Table 3).

## Discussion

The present study utilized an innovative parcellation algorithm that allows for superior parcellation based on deep network methods [20]. Applying this recent parcellation algorithm to the cerebellum, a region often impacted by MS that results in a range of motor and cognitive impairments, is crucial to advance our understanding of this complex brain structure and guide the development of targeted intervention strategies for cerebellar dysfunction in PwMS. Our study aimed to evaluate relationships between cerebellar volumes and measures of motor and cognitive function using an advanced parcellation algorithm in PwMS and HCs. PwMS performed significantly worse on functional measures compared to HC (Table 1). Additionally, functional performance on motor and cognitive tasks was associated with volumetric differences in the cerebellum, underscoring its role in both motor and cognitive processes (Figure 1, Table 2). Finally, we found significant differences in volumetric measures in the corpus medullare, lobule VI, vermis IX, and vermis X between MS fallers and MS non-fallers, indicating volume loss in these regions may be instrumental in predicting falls within MS subjects specifically (Table 3).

Prior studies show that falling is a common result of impairment in MS that is influenced by many factors within motor and cognitive domains [47]. We hypothesized that fallers would perform worse on measures of motor and cognitive function than non-fallers, and that fallers will have lower cerebellar volume than non-fallers globally as well as in motor and cognitive areas. We found significant differences between MS fallers and MS non-fallers on clinical measures such as the SDMT, SF-36 mental, and MSQoL mental, physical, and fatigue subdomains. In volumetric measures, MS fallers showed a significantly lower volume at the corpus medullare, lobule VI, vermis IX, and vermis X compared to MS non-fallers (Table 3, Supplementary Table 1). Previous research has shown reduced gray and white matter cerebellar volumes in PwMS who have fallen when compared with non-faller PwMS [3], supporting our finding. Surprisingly, no motor measures were significantly different between fallers and non-fallers. Our past work suggests that dual-task and backwards walking may better differentiate fallers from non-fallers when compared to standard forward walking and balance measures [48–49], and that the addition of imaging to functional performance may improve fall prediction [10].

A significant finding of the study was that across a majority of the parcellated regions, there were no significant differences in cerebellar volume between PwMS and HCs, which aligns with our hypothesis, and is consistent with prior research [10, 50]. Considering that our sample had a low average disability (average EDSS = 3.8), this was perhaps not surprising as cerebellar atrophy has been noted with disease progression [51–52]. Nevertheless, we did note significant differences between pwMS and HCs in volumes of the corpus medullare, lobules I-III, and lobule V (Supplementary Table 1), which was largely driven by MS fallers.

Within the full sample of PwMS and HCs, we noted significant relationships between cerebellar lobule volumes and functional performance in motor and cognitive domains (Table 2). Importantly, commonly labeled “motor lobules” and “cognitive lobules” were related to performance in both motor and cognitive domains. These findings align with our prior work [10] and highlight the importance of the cerebellum to motor and cognitive control in both healthy adults and PwMS, and the overlap in these areas [14, 16]. We found significant relationships between lower volumes in lobule IX and worse motor performance; recent work has implicated lobule IX in the dorsal attention network [53], suggesting that attention may contribute to motor task performance. Further, some debate exists about the function of lobule VI. In the current study, lobule VI was classified as a cognitive lobule, but has been previously classified as both a motor and non-motor lobule [54]. Interestingly, volumes of the individual cognitive lobules and the total cognitive lobules sum demonstrated a greater number of relationships with functional performance compared to the motor lobules. As previously noted, our MS sample was relatively low in motor disability (mean EDSS = 3.8), and fallers performed equivalent to non-fallers across all motor measures, yet significantly poorer on the SDMT. This finding highlights the impact of cognition on motor performance and falls. Cognitive impairments, such as slowed processing speed and attention deficits, may disrupt the ability to plan and execute movements effectively and delay reaction times, making it difficult to respond quickly to environmental changes. These cognitive impairments can increase the risk of falls, emphasizing the importance of addressing cognition in fall prevention strategies for PwMS. Our data provides additional support for the relationship between cognition and falls and is in line with previous findings that found an association between cognitive dysfunction and fall status in PwMS [3, 55].

Lastly, we compared information processing speed, as reflected by performance on the SDMT, to lobules VI, VIIB, and crus I-II, which are typically considered as cognitive lobules. Our hypothesis was confirmed SDMT was significantly correlated with volumes of lobule VI (p < 0.001) and crus I (p = *0.017*), but not with lobule VIIB and crus II (Table 2). In addition, we correlated motor lobules with the motor measures, and found significant correlations between the TUG, T25FW, walk velocity, summed strength, SSST, and vibration sensation with lobules I-III, and balance to both lobules I-III and V. Measures such as the TUG and SSST require great balance demands and are complex motor tasks which require input from higher cognitive areas [56], while vibration is used as a proxy measure for proprioception [34]. Other studies have shown the lobules I-III of the cerebellum having a greater influence on sensorimotor representations [17], whereas SSST demonstrates increased demand on coordination, balance, and ease of movement [30]. Interestingly, areas typically considered cognitive lobules showed correlations with motor measures, while areas typically considered motor lobules also demonstrated correlations with the SDMT (Table 2).

Previous studies have shown that the cerebellum contributes to motor and cognitive function in PwMS [10]. Herein, we quantified volumes in cerebellar areas and described relationships among these volumes with cognitive and motor skills, and compared these variables among HCs, MS fallers, and MS non-fallers. Additionally, the results of our study improve our understanding of complex structure-function relationships between cerebellar volumes and motor and cognitive functioning, and our results show increased promise for further detection in cerebellum volume changes in MS.

### Limitations:

We acknowledge our small sample size of 31 PwMS and 29 HCs, and recognize that this study is exploratory, but addressing the important issue of falls. Additionally, in clinical data collection, subjects were asked to identify themselves as a faller (≥1 fall in the past month) or a non-faller (no falls in the past month). Subjects who identified as only falling once were grouped with those who have fallen multiple times over the last month, limiting the discrepancy of collecting falls. Furthermore, relying solely on a binary method of collecting fall data may yield misleading results, failing to capture crucial information regarding the consequences of these incidents. Moving forward, incorporating inquiries about injurious falls or other related outcomes in future studies could provide a more comprehensive understanding. Moreover, the reliance on retrospective falls may be limited due to memory recall deficits that are highly prevalent in PwMS and prevent accurate reporting [7, 57]. The use of ecological momentary assessment devices (PRO-Diary, Fitbit) to capture falls data prospectively is recommended. We also did not include any measures of psychological factors that may contribute to falling (i.e., fear of falling, balance confidence, depression, anxiety). Individuals who experience these psychological symptoms often reduce their mobility and physical activity, leading to physical deconditioning [58], and adopt overly cautious gait [59], thereby compromising balance and increasing the risk of falls. Furthermore, clinical testing measures for functional assessment, such as forward walking and Romberg balance, may not be high enough in task complexity and may have limited sensitivity to detect subtle motor and cognitive deficits that contribute to falls in PwMS [48]. Future studies will include self-report measures of psychological factors that may contribute to falls as well as complex, functional motor and cognitive tasks to capture a wider clinical spectrum of both low and high disability persons with MS. Lastly, the parcellation algorithm does not exclude lesions, if present. Rather, it classifies lesion voxels into the foreground, and would not consistently classify lesions into the 28 regions, making it likely that the lesions would be accounted for as reductions in overall volume of each lobule.

## Conclusion

Using the ACAPULCO parcellation tool, we validated the findings of our prior work, demonstrating that both motor and cognitive lobules of the cerebellum contribute to motor and cognitive performance in PwMS. Further, we built upon our prior work quantifying both functional performance and cerebellar volumes that differentiate MS fallers from non-fallers. Data from this low disability sample shows that functional performance and cerebellar volumes in fallers largely drive the differences seen between PwMS and HCs. Future studies should incorporate a larger sample with higher disability to further examine the utility of cerebellar imaging for predicting falls in PwMS.

## Supplementary Material

1

## Figures and Tables

**Table 1.** Demographics, Functional Measures among individuals with MS, Control, MS Fallers, and MS Non-Fallers

	Healthy Control (n=29)	MS (n=31)	MS Fallers (n=15)	MS Non-Fallers (n=16)
**Age (years)**	50.8 (11.6)	49.48 (11.6)	50.3 (12.6)	48.7 (11.0)
**Sex**	9 M; 20F	12M; 19F	6M; 9F	6M; 10F
**Symptom Duration (years)**	-	12.5 (9.6)	13.9 (11.1)	11.2 (8.1)
**EDSS**	-	3.8 [1.0–6.5]	4.0 [1–6.5]	3.5 [1–6.5]
**TUG (s)**	5.8 (1.1)	7.9 (2.4)*	8.6 (2.7)*	7.2 (2.0)*
**T25FW (s)**	4.1 (0.71)	5.6 (2.3)*	6.4 (2.7)*	4.9 (1.5)*
**Walk Velocity**	2.0 (0.32)	1.6 (0.5)*	1.4 (0.5)*	1.7 (0.6)
**2MWT (m)**	200.8 (32.3)	161.2 (46.4)*	147.2 (44.6)*	173.2 (46.0)
**Summed Strength (pounds)**	303.9 (65.2)	205.3 (95.1)*	206.1 (109.7)*	204.6 (82.9)*
**SSST (seconds)**	6.7 (1.4)	10.3 (4.0)*	11.1 (4.1)*	9.62 (3.9)*
**Balance (#Romberg)**	6.0 (0.0)	4.7 (1.1)*	4.6 (1.1)*	4.75 (1.1)*
**Vibration Sensation Avg (vu)**	2.6 (1.6)	6.2 (3.3)*	6.3 (3.4)*	6.1 (3.2)*
**SDMT**	59.7 (6.0)	47.3 (12.3)*	41.8 (11.7)*+	52.8 (10.6)*
**MSWS-12**	-	43.1 (27.5)	49.7 (23.6)	36.8 (30.1)
**BPI Severity**	0.75 (0.85)	2.1 (2.2)*	2.8 (2.7)*	1.5 (1.6)
**BPI Interference**	0.26 (0.63)	1.8 (2.5)*	2.7 (3.1)*	1.0 (1.5)*
**MSQoL fatigue**	-	45.6 (20.8)	34.2 (16.4)†	55.5 (19.4)
**MSQoL mental**	-	69.5 (21.5)	58.4 (22.4) †	79.9 (14.8)
**MSQoL physical**	-	59.9 (16.2)	52.1 (14.3) †	67.3 (14.5)
**SF-36 mental**	55.3 (5.2)	48.4 (11.1)*	42.1 (11.1)* †	54.2 (7.5)
**SF-36 physical**	51.4 (7.0)	38.3 (9.2)*	35.0 (7.9)*	41.4 (9.6)*

All values are listed as mean (SD). *Expanded Disability Status Scale (EDSS); Timed Up and Go (TUG); Timed 25 Foot Walk (T25FW); Two Minute Walk Test (2MWT); Six Spot Step Test (SSST); vibration units (vu); Symbol Digit Modalities Test (SDMT); Multiple Sclerosis 12-item Walking Scale (MSWS-12); Brief Pain Inventory (BPI); Multiple Sclerosis Quality of Life (MSQoL); Short Form-36 (SF-36)*. Mann-Whitney U tests were used to test significant differences between groups.

* Indicates a significant difference between healthy controls and PwMS;

† indicates a significant difference between MS fallers and MS non-fallers (p < 0.05).

**Table 2.** Relationships among Cerebellar Volumes and Functional Performance in persons with MS and HC.

	Lobules I-III	Lobule IV	Lobule V	Lobule VI	Crus I	Crus II	Lobule VIIB	Lobule VIIIA	Lobule VIIIB	Lobule IX	Lobule X	Motor Lobules	Cognitive Lobules
Motor Measures													
TUG	−**.304**	.027	−.158	−.227	−**.353**	−.207	.094	.070	.108	−**.291**	.039	.034	−**.289**
	**.019**	.839	.233	.084	**.006**	.115	.480	.597	.414	**.025**	.768	.799	**.026**
T25FW	−**.370**	.038	−.125	−**.264**	−**.302**	−.243	−.009	−.070	.082	−**.294**	.066	−.094	−**.333**
	**.004**	.776	.345	**.044**	**.020**	.063	.943	.596	.539	**.024**	.618	.480	**.010**
Walk Velocity	**.266**	−.029	.076	**.419**	**.341**	.294	.073	.084	−.102	**.293**	.005	.067	**.428**
	**.040**	.827	.564	**< .001**	**.008**	.023	.579	.524	.439	**.023**	.968	.611	**< .001**
2MWT	.249	.031	.113	.263	**.337**	.179	−.109	−.089	−.193	**.300**	−.068	−.033	**.284**
	.067	.823	.412	.052	**.012**	.190	.427	.520	.159	**.026**	.621	.814	**.036**
Summed Strength	**.445**	−.023	.227	**.283**	**.335**	.195	.071	.178	.224	**.277**	.226	.247	**.330**
	**< .001**	.863	.081	**.028**	**.009**	.135	.591	.175	.086	**.032**	.083	.057	**.010**
SSST	−**.361**	−.005	−.161	−**.353**	−**.367**	−.262	.000	.118	−.023	−**.281**	−.020	.016	−**.406**
	**.006**	.969	.233	**.007**	**.005**	.049	.998	.381	.866	**.034**	.883	.908	**.002**
Balance	**.297**	−.046	**.309**	**.213**	**.322**	.111	.053	−.144	.091	.156	−.023	.040	.254
	**.021**	.727	**.016**	.103	**.012**	.397	.689	.272	.489	.235	.861	.759	.051
Vibration Sensation	−**.366**	.119	−.250	−.121	−.125	−.022	.004	.040	−.092	−.139	−.004	−.045	−.065
	**.004**	.367	0.054	0.357	0.343	.868	.978	.762	.485	.291	.977	.736	.622
Cognitive Measure													
SDMT	**.343**	.066	.247	**.431**	**.310**	.236	.134	−.047	.072	.181	.087	.121	**.411**
	**.008**	.622	.059	**<.001**	**.017**	.072	.312	.723	.588	.171	.513	.361	**.001**

All values listed Spearman’s rho (p). Bolded and shaded values indicate significance at p<0.05. Timed Up and Go (TUG); Timed 25 Foot Walk (T25FW); Two Minute Walk Test (2MWT). Six Spot Step Test (SSST); Symbol Digit Modalities Test (SDMT); Motor lobules indicates the sum of lobules I-V and VIII); Cognitive lobules indicate the sum of lobules VI-VII and Crus I-II

**Table 3.** Differences in Regional Cerebellar Volumes among MS fallers, MS non-fallers, and HCs

Cerebellar region	Control v. MS Faller	Control v. MS Non-Faller	MS Faller v. MS Non-Faller	MS Faller v. All Non-Faller (MS + Control)
Corpus Medullare	**.013**	.222	.027	**.008**
Lobules I-III	**.024**	.361	.163	**.030**
Lobule IV	.701	.813	.401	.522
Lobule V	**.012**	.107	.401	**.035**
Lobule VI	**.009**	.868	**.041**	**.007**
Crus I	**.046**	.962	.163	**.047**
Crus II	.612	.831	.338	.437
Lobule VIIB	.194	.687	.423	.210
Lobule VIIIA	.496	.148	.470	.878
Lobule VIIIB	.379	.180	.495	.765
Lobule IX	.194	.059	.770	.447
Lobule X	.970	.594	.599	.831
Vermis VI	.701	.906	.999	.791
Vermis VII	.310	.209	.520	.676
Vermis VIII	.240	.803	.119	.135
Vermis IX	.235	.470	**.033**	.082
Vermis X	**.032**	.868	**.017**	**.012**
Motor Lobules (I-V VIIIA-B)	.328	.924	.423	.302
Cognitive Lobules (VI, VIIB, Crus I-II)	**.022**	.758	**.007**	**.006**

All values listed are p-values resulting from Mann-Whitney U tests comparing MS fallers, MS non-fallers, and matched HCs. Significant values (p < 0.05) are indicated with bolding and grey shading.

## Data Availability

The data that support the findings of this study are not openly available due to reasons of sensitivity and are available from the corresponding author upon reasonable request.
